# Comparison of Secondary Prevention Status between Percutaneous Coronary Intervention and Coronary Artery Bypass Patients

**DOI:** 10.5935/abc.20170153

**Published:** 2017-11

**Authors:** Xia-qing Gao, Yanfang LI, Zhi-li Jiang

**Affiliations:** 1 Beijing Anzhen Hospital - Capital Medical University, Beijing - China; 2 Beijing Institute of Heart Lung and Blood Vessel Diseases, Beijing - China

**Keywords:** Percutaneous Coronary Intervention, Coronary Artery Bypass, Myocardial Revascularization, Risk Factors

## Abstract

**Background:**

Data are scarce regarding disparities in cardiovascular risk factor management between patients treated with percutaneous coronary intervention (PCI) and those treated with coronary artery bypass grafting (CABG).

**Objective:**

Whether the goal achievement rates of cardiovascular risk factors were different between PCI and CABG patients.

**Methods:**

We retrospectively reviewed the data retrieved from a clinical record database of patients admitted to Beijing Anzhen Hospital between January 1, 2014, and December 31, 2014, who underwent PCI or CABG.

**Results:**

Compared with the CABG group, low-density lipoprotein cholesterol (LDL-C) < 1.8 mmol/L (28.6% vs. 24.7%; p < 0.01), LDL-C < 2.07 mmol/L (43.5% vs. 39.4%; p < 0.01) and blood pressure (BP) < 140/90 mm Hg (85.6% vs. 77.7%; p < 0.01) goal achievement rates were significantly higher in the PCI group. Compared with patients ≥ 60 years old: patients < 60 years old had better BP < 140/90 mm Hg goal achievement rates (87.7% vs. 84.4%; p < 0.01) in the PCI group, and better fasting blood-glucose (FBG) < 7 mmol/L (79.4% vs.72.0%; p < 0.01) and HbA1c < 7% (79.4% vs. 70.1%; p < 0.01) goal achievement rates in the CABG group. Compared with females: males had better LDL-C < 2.07 mmol/L (24.7% vs. 28.5%; p < 0.01), FBG < 7 mmol/L (71.8% vs.75.2%; p < 0.01) and HbA1c < 7% (70.4% vs. 74.1%; p < 0.01) goal achievement rates in the PCI group.

**Conclusion:**

Patients in the PCI group were generally more likely than those in the CABG group to achieve LDL-C < 1.8 mmol/L and BP goals. The control of cardiovascular risk factors differed between patients ≥ 60 years old and < 60 years old. Female patients were less likely to achieve LDL-C, FBG and HbA1c goals.

## Introduction

In China, the number of patients who undergo coronary revascularization increases with cardiovascular disease outbreaks. Percutaneous coronary intervention (PCI) and coronary artery bypass grafting (CABG) are two major coronary revascularization procedures. Although PCI and CABG have saved plenty of lives, they do not prevent the progression of arterial atherosclerosis, and major events and secondary revascularization rates remain high in patients 5 years later.^1^ Taking secondary preventive drugs is important for those patients.^2,3^

Roughly 14,000 patients underwent CABG or PCI in Beijing Anzhen Hospital every year. However, in practice, we found that cardiovascular physicians and cardiothoracic surgeons have different concerns with respect to long-term prognosis, which might influence the prescription of secondary preventive drugs and further leads to unbalanced control of coronary artery disease (CAD)-related risk factors, such as LDL-C, blood pressure (BP), fasting blood-glucose (FBG), and hemoglobin A1c (HbA1c), in PCI and CABG patients. In addition, previous studies have reported that the control of cardiovascular risk factors was different in different age groups and different sex. We hypothesized that patients who have undergone CABG would be less likely than patients who have undergone PCI to achieve lipid, FBG, HbA1c and BP goals. We assessed the goal attainment and clinical outcomes in PCI and CABG patients, and the goal achievement rates in patients ≥ 60 years old and < 60 years old, females and males.

## Methods

### Source population

This retrospective study enrolled 14,230 patients who underwent PCI (n = 9,866) or CABG (n = 4,364) in Beijing Anzhen Hospital between January 1, 2014, and December 31, 2014. The index date was that of the revascularization procedure. We excluded patients (n = 7,707) aged < 18 years with a history of coronary revascularization, malignant tumor, multiple organ dysfunction syndrome, or organ transplantation, without complete demographic data, without continued drug prescription record, or whose second lipid level values were not available after the index date. A total of 6,523 patients were ultimately included in the analysis and matched by propensity score.

### Data collection

Clinical information was retrieved from computerized clinical records, and relevant clinical data were extracted up to December 31, 2015, the start of the data collection period. We obtained the following data: age; sex; history of present illness; comorbidities (hypertension, diabetes, stroke, peripheral vascular disease, chronic kidney disease); cardiovascular disease-related risk factors (smoking, drinking, obesity); coronary artery lesions (SYNTAX score); lipid, BP, FBG and HbA1c levels before discharge and during follow-up. Date of cardiac death, recurrent acute coronary syndrome (ACS), stroke, non-fatal acute myocardial infarction (AMI), and revascularization were also collected for the patient outcome analysis. Composite endpoints were defined as cardiac death, recurrent ACS, and stroke. Recurrent ACS was defined as recurrent non-fatal AMI and unstable angina. Lipid, BP, FBG and HbA1c levels before discharge were defined as lipid, BP, FBG and HbA1c levels before the coronary revascularization procedure, while lipid, BP, FBG and HbA1c levels during follow-up were defined as the most recently lipid, BP, FBG and HbA1c levels (at least 3 months after discharge) if there was no endpoint event, and lipid, BP, FBG and HbA1c levels during re-hospitalization if there was an endpoint event. Hypertension, diabetes, dyslipidemia, stroke, peripheral vascular disease, chronic kidney disease, alcohol heavy drinking, and obesity were defined as published previously.^4^ The follow-up period of each individual from the discharge date until December 31, 2015, was also calculated. Lipid goal attainment was defined as an LDL-C < 1.8 mmol/L (70 mg/dL) and non-high-density lipoprotein cholesterol (HDL-C) < 2.6 mmol/L (100 mg/dL),^5^ or LDL-C < 2.07 mmol/L (80 mg/dL) and non-HDL-C < 2.8 mmol/L (110 mg/dL).^6^ The FBG goal attainment was defined as FBG < 7.0 mmol/L; HbA1c < 7%. Blood pressure goal attainment was defined as BP < 140/90 mm Hg.^7^

This study was approved by the Beijing Anzhen Hospital Ethics Committee.

### Statistical methods

Propensity scores were estimated using a multiple logistic regression analysis. PCI and CABG patients were 1:1 matched using the nearest neighbor matching method. Continuous variables with normal distribution were presented as mean ± standard deviation, and those with non-normal distribution were presented as median and interquartile range. Categorical variables were depicted as absolute numbers and percentages. K-S test was used to verify the normality of the data. Continuous variables were compared using the Wilcoxon signed rank test or a paired *t* test, and categorical variables were compared using the chi-square test. Kaplan-Meier survival curves were used to compare the cumulative incidence of composite endpoint events. Cox regression analysis was performed to evaluate the influence of baseline covariates on composite outcomes. The log-rank test was performed before Cox regression. Variables with P values ≤ 0.10 were candidates for the multivariate model. Covariates included in Cox regression analysis were as follows: age, sex, hypertension, diabetes mellitus, dyslipidemia, smoking, stroke, peripheral artery disease, chronic kidney disease, body mass index (BMI), left ventricular ejection fraction (EF), SYNTAX score, and achievement of LDL-C, FBG, HbA1c and BP goals. All analyses were performed with SPSS (version 22.0; IBM, Armonk, NY, USA). All tests were two-tailed, and P values < 0.05 were considered statistically significant.

## Results

### Baseline characteristics

A total of 6,523 (PCI = 4,728; CABG = 1,795) patients were enrolled in the study. Compared to patients in the PCI group, those in the CABG group were older and more likely to have a history of diabetes and stroke; less likely to have a history of hypertension, and dyslipidemia; and presented lower BMI, HDL-C level and left ventricular EF, and higher SYNTAX score. A total of 1,790 matched patient pairs were created after propensity-score matching was performed for the entire population. The baseline characteristics did not differ significantly between the PCI and CABG groups after the propensity-score matching (Table 1).

**Table 1:** Baseline characteristics of patients in PCI and CABG groups

	Total population			Propensity-matched population		
	PCI n = 4728	CABG n = 1795	p value	PCI n = 1790	CABG n = 1790	p value
Age (years)	58.9 ± 10.2	61.9 ± 9.0	< 0.01	62.0 ± 9.9	61.9 ± 9.0	0.68
Sex (male)	3499(74.0)	1353(75.4)	0.26	1369(76.5)	1349(75.4)	0.43
Hypertension	2394(61.2)	1073(59.8)	< 0.01	1068(59.6)	1072(59.9)	0.87
Diabetes	1461(30.9)	634(35.3)	0.001	632(35.3)	630(35.5)	0.94
Dyslipidemia	3749(79.3)	1361(75.8)	0.002	1360(76.0)	1348(75.3)	0.64
Stroke	265(5.6)	169(9.4)	< 0.01	150(8.4)	168(9.4)	0.29
PVD	52(1.1)	23(1.3)	0.54	27(1.5)	23(1.3)	0.57
CKD	33(0.7)	9(0.5)	0.38	7(0.4)	9(0.5)	0.62
Smoking	2392(50.6)	863(48.1)	0.07	848(47.4)	863(48.2)	0.62
BMI	26.5 ± 3.4	25.4 ± 2.9	< 0.01	25.3 ± 3.3	25.4 ± 2.9	0.57
LVEF (%)	62.2 ± 8.3	60.4 ± 9.0	< 0.01	60.7 ± 9.1	60.5 ± 9.0	0.50
SYNTAX score	23.4 ± 9.3	28.1 ± 10.1	< 0.01	27.8 ± 9.3	28.0 ± 10.2	0.19
**Lipid levels before discharge (mmol/L)**						
TC	4.58 ± 0.9	4.57 ± 1.1	0.87	4.56 ± 1.0	4.57 ± 1.1	0.82
TG	1.87 ± 1.2	1.83 ± 1.1	0.24	1.83 ± 1.1	1.83 ± 1.1	0.99
LDL-C	2.86 ± 0.8	2.88 ± 0.9	0.52	2.87 ± 0.8	2.88 ± 0.9	0.75
HDL-C	1.00 ± 0.2	0.97 ± 0.2	< 0.01	0.97 ± 0.2	0.97 ± 0.2	0.56
**FBG (mmol/L) and HbA1c (%) levels before discharge**						
FBG	6.08 ± 1.7	5.77 ± 1.5	0.07	5.91 ± 1.6	5.77 ± 1.5	0.36
HbA1c	5.93 ± 1.1	5.78 ± 1.1	0.17	5.80 ± 1.1	5.78 ± 1.1	0.64
**Blood pressure (mmHg) before discharge**						
SBP	127.65 ± 15.4	124.04 ± 17.7	0.13	124.5 ± 16.3	124.04 ± 17.7	0.91
DBP	76.54 ± 11.3	75.35 ± 10.6	0.04	75.04 ± 10.3	75.35 ± 10.6	0.32

Values are presented as mean±standard deviation and median with interquartile range or n (%); PCI: percutaneous coronary intervention; CABG: coronary artery bypass grafting; PVD: peripheral vascular disease; CKD: chronic kidney disease; BMI: body mass index; LVEF: left ventricular ejection fraction; TC: total cholesterol; TG: triglyceride; LDL-C: low density lipoprotein cholesterol; HDL-C: high density lipoprotein cholesterol; FBG: fasting blood-glucose; HbA1c: hemoglobin A1C; SBP: systolic blood pressure; DBP: diastolic blood pressure.

### LDL-C, FBG, HbA1c, and BP goal attainment rates in total and propensity matched PCI and CABG patients

Compared with the CABG group, LDL-C < 1.8 mmol/L, LDL-C < 2.07 mmol/L and BP < 140/90 mmHg goal achievement rates in the PCI group were significantly higher in the unmatched patients after discharge. The FBG and HbA1C target attainment rates did not differ significantly between the two groups after discharge (Table 2). In propensity matched patients, LDL-C < 1.8 mmol/L, LDL-C < 2.07 mmol/L and BP < 140/90 mmHg goal achievement rates in the PCI group were significantly higher than in the CABG group. The FBG and HbA1c goal achievement rates were not significantly different between the two groups (Table 2).

**Table 2:** LDL-c, FBG, HbA1c, and BP goal achievement rates in PCI and CABG groups

	Total population			Propensity-matched population		
Risk factor goals	PCI	CABG	p	PCI	CABG	p
LDL-c <1.8 mmol/L^a^	1352(28.6)	443(24.7)	0.002	522(29.2)	442(24.7)	0.003
LDL-c < 2.07 mmol/L^b^	2055(43.5)	708(39.4)	0.003	787(44.0)	707(39.5)	0.007
FBG < 7 mmol/L^c^	3498(74.2)	1342(74.8)	0.492	1361(76.0)	1342 (75.0)	0.46
HbA1c < 7%^c^	3456(73.1)	1321(73.6)	0.686	1349(75.4)	1319(73.7)	0.25
BP < 140/80 mmHg^d^	4049(85.6)	1394(77.7)	0.000	1525(85.2)	1391(77.7)	0.000

Values are presented as n (%);

a ,Chinese guidelines on prevention and treatment of dyslipidemia in adults, 2007;

b ,ESC/EAS guidelines for the management of dyslipidaemias, 2011;

c ,Chinese guidelines on type 2 diabetes prevention and treatment, 2013;

d ,Chinese guidelines on prevention and treatment of hypertension, 2011. PCI: percutaneous coronary intervention; CABG: coronary artery bypass grafting; LDL-C: low density lipoprotein cholesterol; FBG: fasting blood-glucose; HbA1c: hemoglobin A1C; BP: blood pressure.

### Clinical outcomes

**Figure 1:**
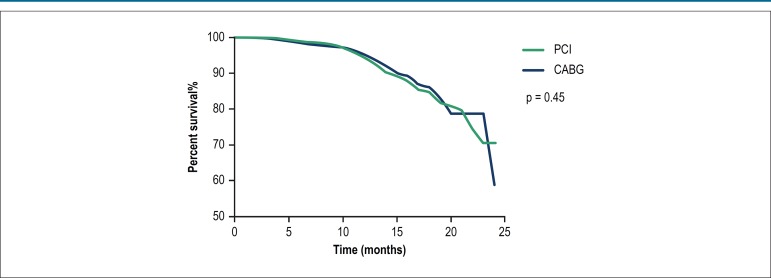
Kaplan-Meier cumulative events for composite endpoint. Composite endpoint events (cardiac death/recurrent acute coronary syndrome/stroke) rate were not significantly different between percutaneous coronary intervention (PCI) and coronary artery bypass grafting (CABG) patients.

**Figure 2:**
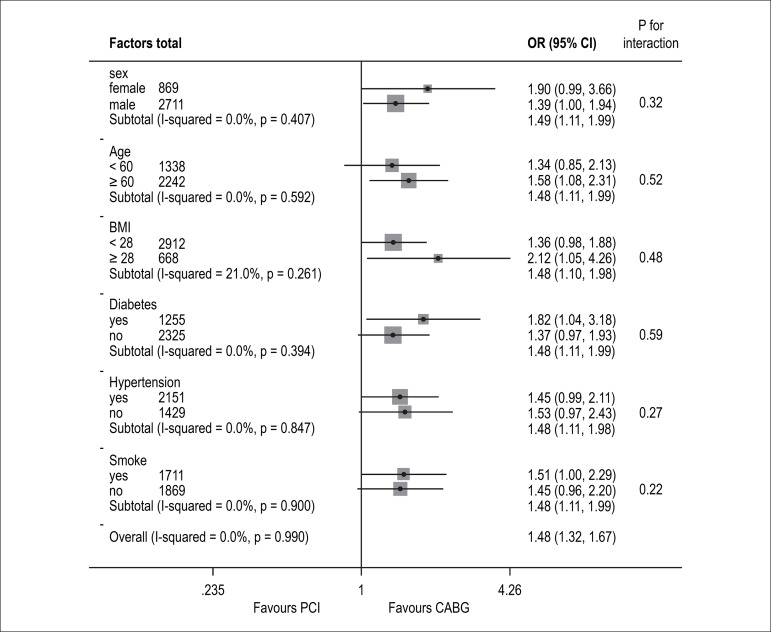
Comparative unadjusted hazard ratios of recurrent ACS for subgroups in propensity-matched populations of the percutaneous coronary intervention (PCI) and coronary artery bypass grafting (CABG) groups. CI: confidence interval; BMI: body mass index; ACS: acute coronary syndrome.

In unmatched patients, composite endpoint rates were significantly higher in the PCI group than in the CABG group (Table 4). The median follow-up duration was 10.99 months. In propensity matched patients, composite endpoint rates were not significantly different between the two groups (Figure 1, Table 4). Recurrent ACS rates were significantly higher in the PCI group than in the CABG group in both matched and unmatched patients (Table 4). Stroke incidence was significantly higher in the CABG group than in the PCI group (Table 4). On multivariable Cox regression analysis, LDL-C < 1.8 mmol/L and HbA1c < 7% were independent predictors of composite endpoints in the unmatched overall, PCI, and CABG patients, hazard ratio were reduced in those patients who achieved goals (Table 3). To determine whether the composite endpoint rates in the matched patients according to PCI and CABG were consistent, we applied subgroup analysis. Compared with patients in the PCI group, patients in the CABG group had better clinical outcome regarding diabetes and obesity, and patients ≥ 60 years old subgroups (Figure 2).

**Table 3:** Independent predictors of composite endpoints in PCI and CABG groups

	Overall			ICP			CRM		
Variables	HR	95% CI	p	HR	95% CI	p	HR	95% CI	p
Sex^a^	0.298	(1.08-1.68)	0.008	0.251	(1.01-1.64)	0.043	0.414	(0.87-2.64)	0.144
PCI vs CABG	0.821	(1.81-2.85)	0.000						
Smoking^a^	1.692	(1.29-2.72)	0.000	1.783	(1.43-3.13)	0.000	1.113	(0.98-1.81)	0.754
LDL-c < 1.8	-2.197	(0.07-0.17)	0.000	-2.329	(0.06-0.16)	0.000	-1.023	(0.09-0.45)	0.000
HbA1c < 7%	-0.363	(0.58-0.85)	0.000	-0.403	(0.54-0.82)	0.000	-0.392	(0.53-0.88)	0.000
EF < 40%^b^	-0.241	(0.52-1.19)	0.252	-0.101	(0.56-1.47)	0.686	-0.825	(0.20-0.95)	0.037
Dyslipidemia^c^	1.164	(0.96-1.45)	0.120	1.256	(1.03-1.63)	0.030	1.09	(0.59-1.43)	0.679
BP < 140/80 mmHg	-0.475	(0.32-0.49)	0.000	-0.432	(0.37-0.50)	0.000	-0.129	(0.39-1.76)	0.788

Values are presented as n (%); CI: confidence interval; HR: Hazard ratio;

a sex and smoke were significant predictors in overall and PCI-treated patients;

b EF > 40% was a significant predictor in CABG-treated patients;

c dyslipidemia was a significant predictor in PCI-treated patients. PCI: percutaneous coronary intervention; CABG: coronary artery bypass grafting; LDL-C: low density lipoprotein cholesterol; HbA1c: hemoglobin A1C; EF: ejection fraction; BP: blood pressure.

**Table 4:** Clinical outcomes in PCI and CABG groups

	Total population				Propensity-matched population			
	PCI	CABG	HR(95% CI)	p	PCI	CABG	HR(95% CI)	p
Composite endpoints	424(9.0)	101(5.6)	1.652(1.32.2.07)	0.000	126(7.0)	101(5.6)	1.27 (0.97.1.66)	0.09
Recurrence ACS	389(8.2)	80(4.5)	5.935(4.619.7.626)	0.000	116(6.5)	80(4.5)	1.48(1.11.1.99)	< 0.01
Stroke	29(0.6)	19(1.1)	1.535(0.858.2.748)	0.146	8(6.4)	19(1.1)	0.42(0.18.0.96)	0.03
Cardiac death	6(0.1)	2(0.1)	3.007(0.606.14.917)	0.157	2(0.1)	2(0.1)	1.00(0.14.7.11)	1.00

Values are presented as n (%). Composite end points included recurrent ACS, stroke and cardiac death. ACS: acute coronary syndrome. PCI: percutaneous coronary intervention.

### LDL-C, FBG, HbA1c, and BP goal attainment rates in unmatched patients with different ages

In unmatched overall and PCI patients, compared with patients ≥ 60 years old: patients < 60 years old had better BP < 140/90 mm Hg goal achievement rates and worse LDL-C < 2.07 mmol/L goal achievement rates. The LDL-C < 1.8 mmol/L, FBG < 7 mmol/L, and HbA1c < 7% goal achievement rates were not significantly different. In unmatched CABG patients, compared with patients ≥ 60 years old: patients < 60 years old had better FBG < 7 mmol/L, HbA1c < 7%, BP < 140/90 mm Hg goal achievement rates, the LDL-C < 1.8 mmol/L and LDL-C < 2.07 mmol/L goal achievement rates were not significantly different between the two groups (Table 5).

**Table 5:** LDL-c, FBG, HbA1c, and BP goal achievement rates in different age and sex

	Overall			PCI			CABG		
LDL-c, FBG, HbA1c, and BP goal achievement rates in patients who < 60 and ≥ 60 year old									
Risk factor goals	< 60	≥ 60	p	< 60	≥ 60	p	< 60	≥ 60	p
LDL-c < 1,8 mmol/L^a^	640(26.3)	1155(28.3)	0.079	474(27.0)	878(29.6)††	0.056	166(24.4)	277(24.8)	0.859
LDL-c < 2,07 mmol/L^b^	967(39.7)	1796(44.0)	0.001	703(40.0)	1352(45.5) ††	0.001	264(38.9)	444(39.8)	0.704
FBG < 7 mmol/L^c^	1817(74.5)	3023(74.0)	0.608	1278(72.8)	2219(74.7)	0.138	539(79.4)‡‡	804(72.0)	0.001
HbA1c < 7%c	1805(72.9)	2972(72.7)	0.240	1266(72.0)	2190(73.7) †	0.196	539(79.4) ‡‡	782(70.1)	0.001
BP < 140/80 mmHg^d^	2093(85.9)	3350(82.0)	0.000	1541(87.7)*	2508(84.4) ††	0.002	552(81.3)	842(75.4)	0.004
**LDL-c, FBG, HbA1c, and BP goal achievement rates in female and male**									
**Risk factor goals**	**Female**	**Male**	**p**	**Female**	**Male**	**p**	**Female**	**Male**	**p**
LDL-c < 1,8 mmol/L^a^	399(24.7)	1396(28.5)	0.003	306(25.5)	1046(29.6)	0.006	93(22.2)	350(25.4)	0.188
LDL-c < 2,07 mmol/L^b^	661(40.9)	2102(42.8)	0.165	502(41.9)	1553(44.0)	0.197	159(38.0)	549(39.9)	0.502
FBG < 7 mmol/L^c^	1152(71.8)	3689(75.2)	0.002	851(71.0)	2647(75.0)	0.006	301(72.0)	1042(75.7)	0.131
HbA1c < 7%c	1139(70.4)	3638(74.1)	0.003	832(69.4)	2624(74.4)	0.001	307(73.4)	1014(73.6)	0.937
BP < 140/80 mmHg^d^	1330(82.3)	4113(83.8)	0.137	1019(85.0)	3030(85.9)	0.457	311(74.4)	1083(78.6)	0.068

Values are presented as n (%);

a Chinese guidelines on prevention and treatment of dyslipidemia in adults, 2007;

b ESC/EAS guidelines for the management of dyslipidemia, 2011;

c Chinese guidelines on type 2 diabetes prevention and treatment, 2013;

d d Chinese guidelines on prevention and treatment of hypertension, 2011;

* in patients who < 60, compared with CABG group, p < 0.01;

†† in patients who ≥ 60, compared with CABG group, p < 0.01;

† in patients who ≥ 60, compared with CABG group, p < 0.05;

‡‡ in patients who < 60, compared with PCI group, p < 0.01. PCI: percutaneous coronary intervention; CABG: coronary artery bypass grafting; LDL-C: low density lipoprotein cholesterol; FBG: fasting blood-glucose; HbA1c: hemoglobin A1C; BP: blood pressure.

### LDL-C, FBG, HbA1c, and BP goal attainment rates in unmatched patients of different sexes

In unmatched overall and PCI patients, compared with females: males had better LDL-C < 1.8 mmol/L, FBG < 7 mmol/L, and HbA1c < 7% goal achievement rates. The LDL-C < 2.07 mmol/L and BP < 140/90 mmHg goal achievement rates were not significantly different. Those goal achievement rates were not significantly different in CABG patients between females and males (Table 5).

### LDL-C, FBG, HbA1c, and BP goal attainment rates in unmatched patients with different ages and different sexes

In unmatched patients ≥ 60 years old, compared with females, males had better LDL-C < 1.8 mmol/L, FBG < 7 mmol/L, and HbA1c < 7% goal achievement rates. The LDL-C < 2.07 mmol/L and BP < 140/90 mmHg goal achievement rates were not significantly different. Those goal achievement rates were not significantly different in patients < 60 years old between females and males (Table 6).

**Table 6:** LDL-c, FBG, HbA1c, and BP goal achievement rates between different sex in patients < 60 years old and patients ≥ 60 years old

Risk factor goals	< 60			≥ 60		
	Female	Male	p	Female	Male	p
LDL-c < 1,8 mmol/La	81(24.5)	559(26.5)	0.426	318(24.7)	837(29.9)	0.001
LDL-c < 2,07 mmol/Lb	120(36.3)	847(40.2)	0.171	541(42.1)	1255(44.8)	0.100
FBG < 7 mmol/Lc	239(72.2)	1578(74.9)	0.290	913(71.0)	2111(75.4)	0.003
HbA1c < 7%c	232(70.1)	1573(74.7)	0.076	907(70.5)	2065(73.8)	0.032
BP < 140/80 mmHgd	277(83.7)	1816(86.2)	0.217	1053(81.9)	2297(82.0)	0.905

Values are presented as n (%). LDL-C: low density lipoprotein cholesterol; FBG: fasting blood-glucose; HbA1c: hemoglobin A1C; BP: blood pressure

## Discussion

PCI and CABG techniques were developed rapidly in the late 90s in China. The surgical volume of PCI was increased by 30%-50% per year, and up to 567583 in 2015, forefront in the world. With the improvement of surgical techniques, mortality of CABG was reduced greatly, and was acceptable by an increasing number of patients. Although PCI and CABG successfully saved plenty of lives, how to decrease the incidence of revascularization is a major problem at present. Therefore, the emphasis of secondary prevention is particularly important after PCI and CABG.

In the present study, our major findings are as follows: (a) in overall and the propensity score-matched patients, lipid and BP goal attainment rates were different between PCI and CABG patients; however, LDL-C, FBG, HbA1c and BP goal attainment rates were not optimistic in either group, (b) the LDL-C and BP goal achievement rates in the PCI group, and the FBG and HbA1c goal achievement rates in the CABG group were different between patients < 60 years old and those ≥ 60 years old; (c) the LDL-C, FBG and HbA1c goal achievement rates were significantly lower in females in the PCI group, as well as in patients ≥ 60 years old.

LDL-C and BP goal achievement rates in the PCI group were significantly higher than in the CABG group, and a possible reason might be the difference in medication use and adherence. Hlatky et. al have observed that medication possession ratios of secondary preventive drugs were significantly lower in CABG patients than in PCI patients, and the use of statins was significantly lower in CABG patients than in PCI patients.^8^ Possible reasons for such disparities might be as follows: (a) in our hospital, some patients after CABG were taken care of by surgeons, treatment strategies differed between cardiologists and cardiothoracic surgeons. Cardiologists followed guidelines and had better performance in using preventive drugs than cardiothoracic surgeons, while cardiothoracic surgeons usually pay more attention to whether the surgery was successful, postoperative complications and wound repair situations rather than secondary prevention drug prescription and health education before discharge;^4^ (b) some other patients might be followed up by cardiologists after CABG in the outpatient clinic, however, cardiologists may have been trained to consider CABG as a more effective or complete treatment, leading to the neglect of long-term secondary prevention; and (c) patients might feel that a CABG is the definitive treatment for their CAD and that medications are no longer necessary, making them less likely to visit doctors in outpatient clinics and take useful suggestions from them.^9^ The FBG and HbA1c goal achievement rates were low and were not significantly different between the PCI and the CABG group. Only almost less than one third of all diabetic patients achieved their FBG and HbA1c goals. Hypoglycemic drugs do not belong to the optimal medical therapy (OMT) drugs, sometimes the cardiologists just focused on the OMT treatment and ignored the FBG control; another reason might be that diabetic patients were recommended to go to endocrinology outpatient clinics by cardiologists and cardiothoracic surgeons, but these patients were always less likely to visit another outpatient clinic since they thought they already had one.

In spite of the disparities between PCI and CABG patients in cardiovascular risk factor control, the achievement rates of LDL-C, FBG, HbA1c and BP goals remain low in the PCI group. Possible reason might be that interventional cardiologists are usually more conditioned to consider dual antiplatelet therapy (DAPT) issues and sometimes ignore the use of other secondary prevention drugs.

In our study, composite endpoints were significantly higher in the un-matched PCI group than in the CABG group. This was consistent with previous studies which suggested that patients who underwent CABG had better clinical outcomes than those who underwent PCI.^10,11^ In the propensity-matched patients, although the recurrent ACS rate was significantly higher in the PCI group, composite endpoints were not significantly different between the two groups. In our multivariate Cox regression analysis, sex, smoking, and achieved LDL-c, HbA1c, and BP goals were independent predictors for composite endpoints in PCI patients, while EF>40%, achieved LDL-c, and BP goals were independent predictors for composite endpoints in CABG patients. The LDL-c and BP goal achievement rates were significantly higher in the PCI group, the HbA1c target attainment rate, although not significantly different, was better in the propensity matched PCI group. The results suggested that secondary prevention was important in reducing post-revascularization events. In the propensity matched subgroup analysis, patients with diabetes, obesity, and ≥ 60 years old had better clinical outcome in the CABG group, in accordance with former studies.^12-14^

The LDL-C < 2.07 mmol/L goal attainment rate of ACS patients in the DYSIS-China study was 29.7%. In our study, it was significantly improved, but remain very low in PCI and CABG patients. Baseline LDL-C levels were reported to be lower in Chinese ACS patients than in western countries' ACS patients in previous studies.^15,16^ LDL-C was recommended to be lower than 2.07 mmol/L in Chinese lipid management guideline. Whether the target LDL-C should be in accordance with that of western countries lipid management guidelines (LDL-C < 1.8 mmol/L) remains controversial. Lee et al. have observed that, compared with LDL-C < 2.6 mmol/L, an LDL-C < 1.8 mmol/L did not improve survival in ACS patients.^17^ However, in our study, achieving the LDL-C < 1.8 mmol/L goal was an independent predictor of decreased composite endpoint risk,^18^ which suggests that the LDL-C goal of Chinese lipid management guideline in the future should be consistent with that of western countries.

In the PCI group, BP goal achievement rate was higher in patients < 60 years old than those ≥ 60 years old, the FBG and HbA1c goal achievement rates were higher in patients < 60 years old in the CABG group. The results were consistent with those of previous studies that older patients always underuse the recommended secondary preventive drugs and always had bad adherence to those drugs,^19^ which further lead to worse risk factor target attainment. However, the LDL-C goal achievement was much better in patients ≥ 60 years old. The result differed from most of the former studies, but was consistent with that of Rajendran et al.,^20^ who found that older patients more often achieved lipid target than younger patients. The results may suggested that the clinicians in our hospital are realizing the importance of statin treatment with each passing day. Hogh et al.,^21^ have discovered that age-related differences in using secondary prevention drugs have been reduced or even eliminated, which suggested that the disparities in risk factor target attainment will also be eliminated over time. Why the risk factor target attainment was inconsistent between PCI and CABG in different age groups remains unclear, but the results suggested that we should pay more attention to older patients in secondary prevention.

Females were considered to be less likely to achieved their cardiovascular risk factor targets since they were less likely to take secondary preventive drugs due to many reasons. For example, the lowering estrogen levels, higher adverse events and poor adherence might have influence on drug use.^22^ However, in the study by Jankowski et al.,^23^ they have found that the frequency of achieving recommended goals in secondary prevention were not sex-related. In our study, the LDL-C, FBG, and HbA1c goal achievement rates were significantly higher in males than in females in the PCI group and in patients ≥ 60 years old. The result suggested that we should pay attention to older women during the secondary prevention process and make sure they are given the optimal treatment.

### Limitations of the study

Our study had several limitations. Firstly, it was a single-center observational study performed at a major cardiovascular hospital in China, and the clinical strategies of physicians and surgeons may differ from those of other hospitals in China. Secondly, although propensity score matching was performed to adjust for potential confounding factors in PCI and CABG patients, initial selection bias and unmeasured variables exist.

## Conclusion

Our research showed that there are disparities between PCI and CABG patients in CAD-related risk factor target attainment. Secondary prevention is critical in reducing post-revascularization endpoints. The risk factor target attainment also differed between patients ≥ 60 years old and < 60 years old, females and males, which suggested that cardiologists and cardiothoracic surgeons should pay more attention to those special patients and make correct clinical decisions in the secondary prevention process, which can further ensure those patients have a better prognosis and greater clinical benefits.
